# Purinergic Calcium Signals in Tumor-Derived Endothelium

**DOI:** 10.3390/cancers11060766

**Published:** 2019-06-01

**Authors:** Giorgia Scarpellino, Tullio Genova, Daniele Avanzato, Michela Bernardini, Serena Bianco, Sara Petrillo, Emanuela Tolosano, Joana Rita de Almeida Vieira, Benedetta Bussolati, Alessandra Fiorio Pla, Luca Munaron

**Affiliations:** 1 Department of Life Sciences and Systems Biology, University of Torino, via Accademia Albertina 13, 10123 Torino, Italy; giorgia.scarpellino@unito.it (G.S.); tullio.genova@unito.it (T.G.); michela.bernardini@hotmail.com (M.B.); joanarita@live.com.pt (J.R.d.A.V.); alessandra.fiorio@unito.it (A.F.P.); 2 Department of Surgical Sciences, University of Torino, via Nizza 230, 10126 Torino, Italy; 3 Department of Oncology, University of Torino, 10060 Torino, Italy; daniele.avanzato@unito.it; 4 Department of Public Health and Pediatrics, University of Torino, 10126 Torino, Italy; serena.bianco@unito.it; 5 Department of Molecular Biotechnology and Health Sciences, University of Torino, Via Nizza 52, 10126 Torino, Italy; sara.petrillo@unito.it (S.P.); emanuela.tolosano@unito.it (E.T.); benedetta.bussolati@unito.it (B.B.)

**Keywords:** tumor-derived endothelial cells, endothelium, purinergic signaling, purinergic receptors, ion channels, calcium signaling, tumor angiogenesis, migration

## Abstract

Tumor microenvironment is particularly enriched with extracellular ATP (eATP), but conflicting evidence has been provided on its functional effects on tumor growth and vascular remodeling. We have previously shown that high eATP concentrations exert a strong anti-migratory, antiangiogenic and normalizing activity on human tumor-derived endothelial cells (TECs). Since both metabotropic and ionotropic purinergic receptors trigger cytosolic calcium increase ([Ca^2+^]c), the present work investigated the properties of [Ca^2+^]c events elicited by high eATP in TECs and their role in anti-migratory activity. In particular, the quantitative and kinetic properties of purinergic-induced Ca^2+^ release from intracellular stores and Ca^2+^ entry from extracellular medium were investigated. The main conclusions are: (1) stimulation of TECs with high eATP triggers [Ca^2+^]c signals which include Ca^2+^ mobilization from intracellular stores (mainly ER) and Ca^2+^ entry through the plasma membrane; (2) the long-lasting Ca^2+^ influx phase requires both store-operated Ca^2+^ entry (SOCE) and non-SOCE components; (3) SOCE is not significantly involved in the antimigratory effect of high ATP stimulation; (4) ER is the main source for intracellular Ca^2+^ release by eATP: it is required for the constitutive migratory potential of TECs but is not the only determinant for the inhibitory effect of high eATP; (5) a complex interplay occurs among ER, mitochondria and lysosomes upon purinergic stimulation; (6) high eUTP is unable to inhibit TEC migration and evokes [Ca^2+^]c signals very similar to those described for eATP. The potential role played by store-independent Ca^2+^ entry and Ca^2+^-independent events in the regulation of TEC migration by high purinergic stimula deserves future investigation.

## 1. Introduction

Tumor microenvironment is characterized by the accumulation of ATP and other nucleotides, reaching concentrations much higher than those measured in healthy tissues [1,2,3,4]. The total amount of extracellular ATP (eATP) depends on the balance between its release by different cell types and its breakdown into ADP and adenosine (ADO) by ectonucleotidases [5,6,7,8]. In particular, tumor-associated eATP derives from necrotic and inflammatory cells, but it can also be released directly from cancer cells [2,3,9]. eATP acts as a signaling molecule and, together with other related nucleotides and nucleosides, participates in the purinergic signaling upon binding with purinergic receptors on the cells surface [10]. Purinoceptors are classified in two major families: metabotropic P1 receptors (P1Rs) and P2 receptors (P2Rs), which are further divided into ionotropic P2X receptors (P2XRs) and metabotropic P2Y receptors (P2YRs). Moreover, while P1Rs are activated only by Adenosine, P2Rs recognize ATP, ADP, UTP and UDP [11]. The ionotropic P2XRs sub-families are ligand-gated Ca^2+^ permeable ion channels [12] and include seven members (P2X1-7). P2XRs can assemble into homo- or heterotrimeric functional channels, whose preferential physiological agonist is ATP [13]. Instead, the metabotropic P2YRs sub-families are G protein-coupled receptors (GPCRs) which include eight subtypes, further divided into two other groups based on their G protein selectivity and sequence similarity. In particular, P2Y1R, P2Y2R, P2Y4R, P2Y6R and P2Y11R mainly associate with G_q_ protein and activate PLCβ, while P2Y12R, P2Y13R and P2Y14R preferentially couple with G_i/o_, suppressing adenylyl cyclase activity [11]. Nevertheless, P2YRs can also be divided according to the preferential endogenous agonist: ATP for P2Y2R and P2Y11; ADP for P2Y1R, P2Y12R and P2Y13R; UTP for P2Y2R and P2Y4R; UDP for P2Y6R and P2Y14R; UDP glucose for P2Y14R [14]. The activation of PLCβ by P2YRs leads to inositol 1,4,5-trisphosphate (InsP3) and diacylglycerol (DAG) production starting from phosphatidylinositol 4,5-bisphosphate (PIP2). InsP3 is an important intracellular second messenger which, upon binding with InsP3 receptors (IP3R) on endoplasmic reticulum (ER), elicits an increase in cytosolic Ca^2+^ concentration ([Ca^2+^]c) due to the rapid passage of Ca^2+^ content from the ER lumen to the cytosol [15]. The resulting Ca^2+^ release and the subsequent decrease in ER luminal [Ca^2+^] [16,17] trigger the store-operated Ca^2+^ entry (SOCE) mechanism [15]. Stromal Interaction Molecule (STIM) proteins are the ER Ca^2+^ levels sensors that detect the ER Ca^2+^ depletion, cluster at ER-plasma membrane surface and finally activate store-operated channels (SOCs), such as Orai1-3 channels and TRPC1-7 [18]. SOCE is the principal Ca^2+^ signaling pathway responsible for the Ca^2+^ entry modulated by the intracellular stores, both in normal and cancer cells [18,19]. It is triggered by a set of different membrane receptors [20], including P2YRs. Therefore, purinergic stimulation triggers [Ca^2+^]c increase which consist of an initial peak caused by InsP3-dependent Ca^2+^ release from intracellular stores, followed by a sustained plateau phase dependent on Ca^2+^ influx from extracellular medium [21].

Importantly, SOCE mechanisms are involved in the regulation of cellular proliferation, migration, differentiation and apoptosis [22,23], playing important roles in cancer cell migration, metastasis and evasion of apoptosis, as well as in endothelial proliferation and migration [18,24,25].

Nowadays, growing interest has been focused on the therapeutic potential of purinergic signaling since it plays a fundamental role in both physiological and physio-pathological processes. In particular, eATP controls several acute vascular functions [6,7,8,26,27,28], such as angiogenesis, that are mediated by endothelial cell proliferation, migration and death. The potential efficacy of purinergic signaling in the treatment of cancer has recently been evaluated [29] but its functional effects on tumor cells and vasculature in vitro and in vivo are still debated and must be further clarified [3,30,31].

Rapaport and colleagues have demonstrated for the first time an anti-neoplastic activity of eATP. Consistently, recent studies have revealed an antitumor activity of extracellular nucleotides in different types of cancer [30,32]. P2Rs are highly expressed by many tumors and their modulation may play an important role in tumor progression [3,30,31,33]. However, whether they exert a promoting or suppressive effect on tumor’s growth is still a controversial issue and the underlying mechanisms remain partially unknown [34,35].

Furthermore, very few studies on the effects of purinergic stimulation in the tumor endothelium are currently available. The tumor vasculature strongly differs from the vasculature of healthy tissues from both a morphological and functional point of view. A deeper knowledge of the mechanisms underlying the sensitivity of tumor endothelium to high eATP concentrations (which are typical of the tumor microenvironment) could be helpful in this context.

We have recently shown that high non-physiological eATP doses (>20 µM) strongly inhibit migration of ECs isolated from human breast carcinoma (BTEC) and enhance attraction of human pericytes, leading to a decrease of endothelial permeability, a hallmark of vessel normalization [36]. This process could contribute to dampening hypoxia in the tumor microenvironment and to elicit a transition towards a more physiological condition.

In addition to Ca^2+^ signaling, some P2YRs (mainly P2Y11) and P2XRs promote intracellular cAMP increase that can in turn regulate endothelial barrier stabilization and inhibit cell migration [37,38]. Since the intracellular Ca^2+^ increase is one of the universal and major routes for intracellular cAMP release and both P2X7 and P2Y11 are able to trigger cytosolic Ca^2+^ elevation, here we decided to investigate the general properties of Ca^2+^ signaling activated by high eATP on TEC.

## 2. Results

### 2.1. High ATP Stimulation Triggers Biphasic Ca^2+^ Signals in Human Tumor-Derived Endothelial Cells

**Figure 1:**
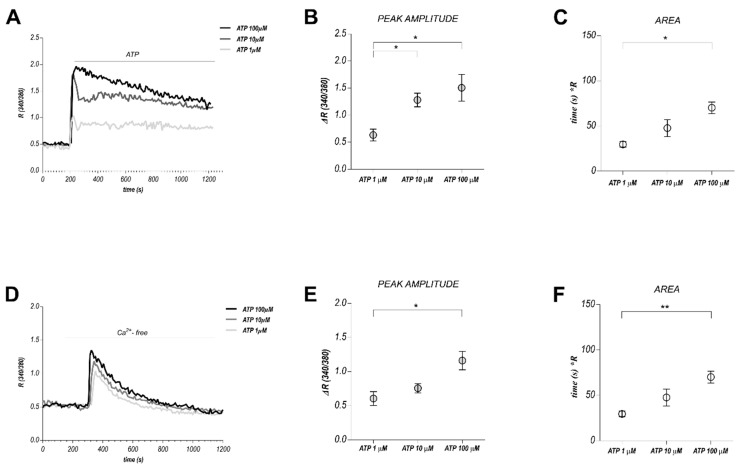
High eATP stimulation triggers biphasic Ca^2+^ signals in BTEC. (**A**) Representative traces of different Ca^2+^ signals evoked by three different eATP concentrations (1, 10, 100 µM). (**B**,**C**) Quantification of the peak amplitude and area obtained upon 1, 10, 100 µM ATP stimulation. Area is calculated at 300 s from the beginning of the response. Data are expressed as mean ± SEM. * *p*-value < 0.05. (**D**) Representative traces obtained upon 100 µM ATP stimulation in Ca^2+^ free extracellular medium. (**E**,**F**) Quantification of peak amplitude and total area under the calcium spike. Data are expressed as mean ± SEM. * *p*-value < 0.05; ** *p*-value < 0.005. Traces in A and D represent the average of all the cells in one representative experiment.

Purinergic stimulation of BTEC with different eATP concentrations (1, 10, 100 μM) evoked [Ca^2+^]c increased in the great majority of cells examined (Figure 1A). An initial Ca^2+^ transient (peak) is usually followed by a sustained long-lasting phase (plateau). Based on the dose-dependent amplitude and area of peak and plateau phases (Figure 1B,C), and according to the previously reported functional effects on BTEC migration [36], the following experiments were carried out treating BTEC with 100 μM ATP. Interestingly, renal tumor-derived endothelial cells (RTEC) (characterized in [39]), displayed a similar biphasic response to ATP (Figure S1A): moreover, their migration, similarly to BTEC, was affected by 100 μM ATP treatment (Figure S1B). When ATP was applied in a calcium-free extracellular solution (0 Ca_out_), only a dose-dependent transient spike was detectable, due to Ca^2+^ release from intracellular stores, while the plateau phase, mainly related to Ca^2+^ entry from the extracellular medium, was completely abolished (Figure 1D–F).

### 2.2. ATP-Evoked Ca^2+^ Influx Includes Both Store Operated Ca^2+^ Entry (SOCE) and Non-SOCE Components

**Figure 2:**
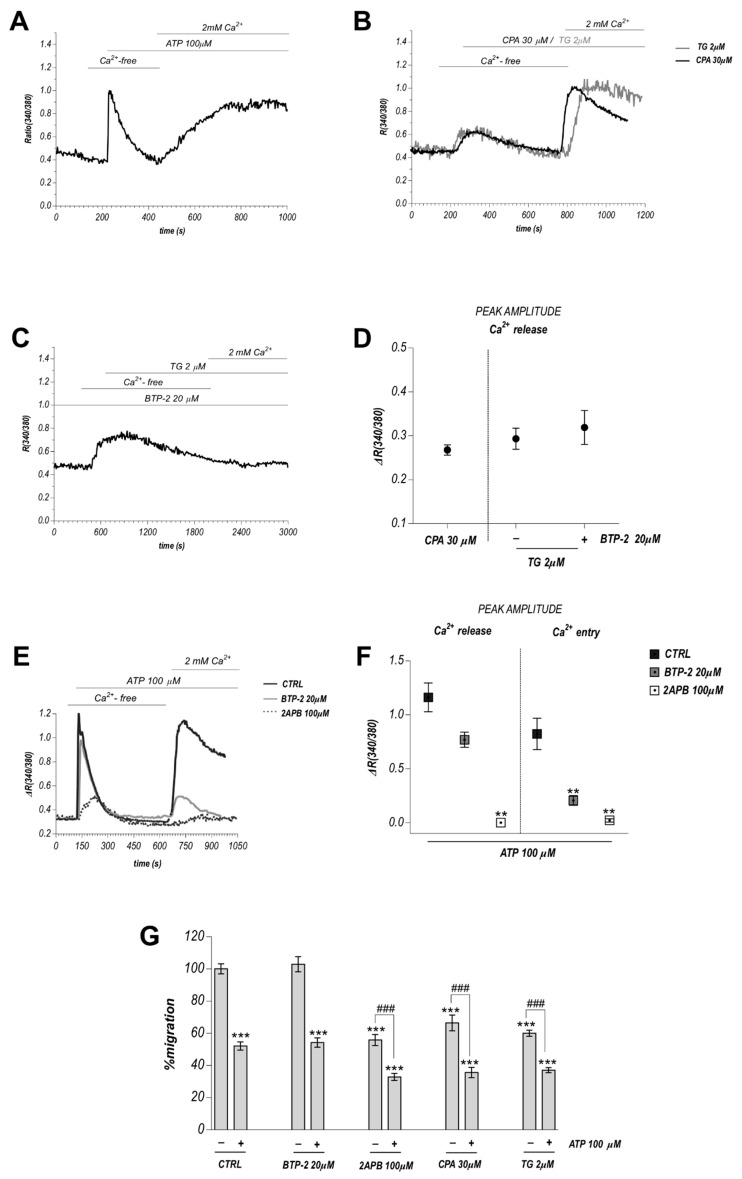
Calcium release, SOCE and relative involvement in anti-migratory action of high ATP. (**A**,**B**) Representative average traces obtained from the application of the ’Ca^2+^ add-back’ protocol upon treatment with 100 µM ATP (**A**), 30 µM CPA or 2 µM TG. (**C**) Preincubation with 20 µM BTP-2 (20 min) completely abolished TG-induced SOCE. Representative average trace. (**D**) Quantification of the previous experiments. Data are expressed as mean ± SEM. (**E**) Effect of preincubation with 20 µM BTP-2 or 100 µM 2-APB on ATP-induced calcium response. Representative average traces. (**F**) Quantification of the previous experiments. Data are expressed as mean SEM. ** *p*-value < 0.005 vs. CTRL. (**G**) BTEC migration evaluated by scratch wound healing measurements. Effects of CPA, TG, BTP-2, 2-APB. Data are expressed as percentage of migration at 8 h, normalized to the corresponding control (CTRL) and expressed as mean ± SEM. Mann-Whitney test: *** *p*-value < 0.0005 vs. CTRL; ### *p*-value < 0.0005.

In order to separate and quantify the Ca^2+^ release from intracellular stores and Ca^2+^ entry from the extracellular medium in response to 100 μM ATP, we applied the ‘Ca^2+^ add-back’ protocol (see Section 4), in which the cells were first stimulated with ATP in 0 Ca_out_, followed by 2 mM Ca^2+^ addition in the continuous presence of the agonist. Figure 2A shows Ca^2+^ entry following the Ca^2+^ release observed in 0 Ca_out_. The expression of a functional SOCE machinery in TEC was unveiled stimulating BTEC with 30 µM Cyclopiazonic acid (CPA) or 2 µM Thapsigargin (TG), two widely used ER depletors and SOCE activators, (Figure 2B: quantification in Figure 2D), two widely used ER depletors and SOCE promoters. Similar results were obtained in RTEC (Figure S1C–E).

### 2.3. SOCE Component Is Not Required for the Anti-Migratory Activity of ATP

A pharmacological approach was employed to modulate the SOCE-related component of the Ca^2+^ response to 100 μM ATP in BTEC and to clarify its contribution to the process under investigation. As expected, pretreatment with the SOCE inhibitor 20 µM BTP-2 (20 min) completely abolished TG-induced calcium entry in add-back experiments (Figure 2C,D).

The same treatment with BTP-2 significantly inhibited ATP-mediated Ca^2+^ entry leaving unaltered the release from the internal stores (Figure 2E,F). A subpopulation of cells showed a BTP-2-insensitive component, suggesting the existence of a non-SOCE component (Figure 2E,F). This observation is in nice agreement with an expression of functional store-independent and calcium-permeable P2RX-related ion channels, previously described in TEC [36]. The inhibition of InsP3 receptors by preincubation with 100 µM 2-APB (5 min) significantly reduced both ATP-induced calcium entry and release (Figure 2E,F).

To evaluate the potential involvement of Ca^2+^ release on BTEC migration, we performed migration assays preincubating the cells with 30 µM CPA or 2 µM TG, and subsequently stimulating in the presence or absence of ATP. Both CPA and TG remarkably reduced BTEC migration resembling the effect of 100 µM ATP: however, co-incubation with both ATP and CPA or TG produced further anti-migratory activity (Figure 2G), showing that the ER-related component is not the only mechanism responsible for the functional effect.

Interestingly, complete inhibition of SOCE by BTP-2 pre-incubation failed to alter the ATP-mediated inhibitory effect on BTEC migration (Figure 2G), suggesting that SOCE does not play a major role in the ATP-mediated inhibitory effect of BTEC migration.

### 2.4. Ca^2+^ Signals Mediated by Other Purinergic Agonists

**Figure 3:**
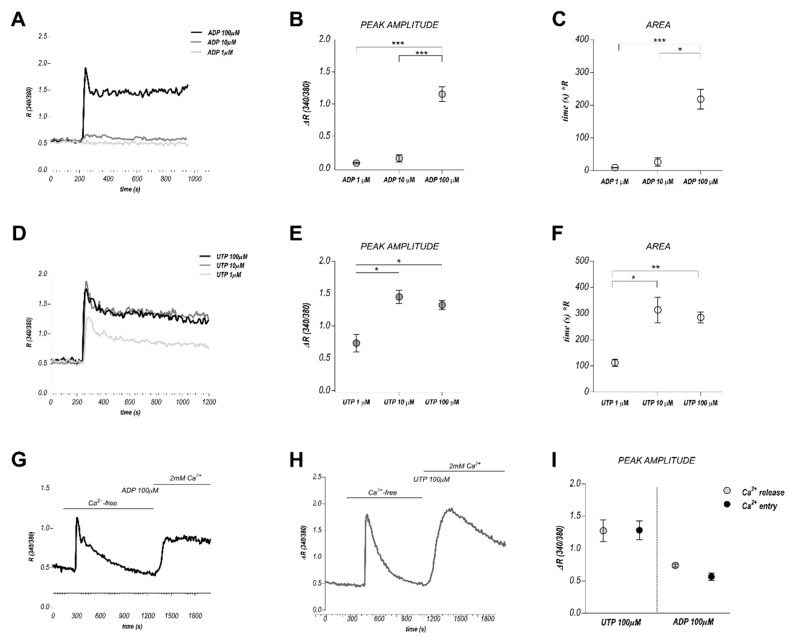
High eADP and eUTP stimulation triggers biphasic Ca^2+^ signals in BTEC. (**A**) Representative traces of different Ca^2+^ signals evoked by three different ADP concentrations (1, 10, 100 µM). (**B**,**C**) Quantification of the peak amplitude and area obtained upon 1, 10, 100 µM ADP stimulation. Area is calculated at 300 s from the beginning of the response. Data are expressed as mean ± SEM. * *p*-value < 0.05; ** *p*-value < 0.005 *** *p*-value < 0.0005. (**D**–**F**) The same for eUTP stimulation. (**G**–**I**) Representative average traces obtained from the application of the ‘Ca^2+^ add-back’ protocol upon treatment with 100 µM ADP or 100 µM UTP and relative quantifications. Data are expressed as mean ± SEM.

We previously reported anti-migratory activity for 100 µM ADP, but not for 100 μM UTP [36]. Both the agonists evoked dose-response calcium increases in physiological extracellular solution (Figure 3A–F). The add-back protocol allowed us to quantify Ca^2+^ release and Ca^2+^ entry (Figure 3G–I).

Moreover, BTEC stimulation with 100 µM Adenosine (ADO), a P1R agonist that inhibits BTEC migration [36], triggered a long lasting calcium signal (Supplementary Figure S2A): this evidence reveals that P1 receptors, expressed both in BTEC and RTEC with a strong prevalence for A2B subtype (see Supplementary Figure S2B), are functional.

### 2.5. Contribution of ER and Other Organelles in the Calcium Release Induced by ATP and UTP

**Figure 4:**
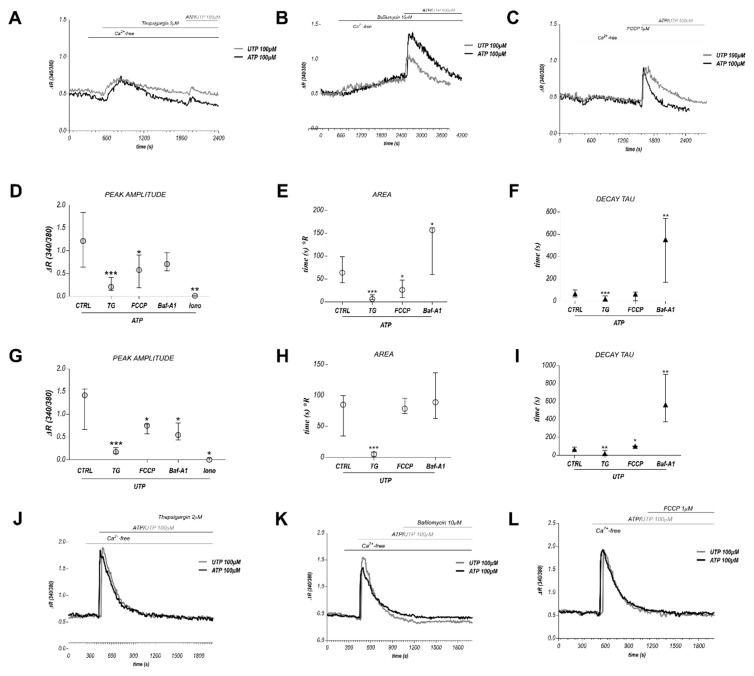
Interplay among different intracellular organelles in BTEC. (**A**–**C**) Effect of 2 µM TG, 10 µM Baf-A1 and 1 µM FCCP on the responses to 100 µM eATP or UTP. Representative traces. (**D**–**I**) Quantification of the previous experiments. Data are expressed as median over the total range of values. * *p*-value < 0.05; ** *p*-value < 0.005; *** *p*-value < 0.0005 vs. CTRL. (**J**–**L**) Effect of TG, Baf-A1 and FCCP in cells pretreated with 100 µM ATP or UTP.

Finally, we decided to compare the calcium store dynamics involved in the response to 100 µM ATP, a potent inhibitor of BTEC migration, to the response to 100 µM UTP, a purinergic agonist that failed to exert the same functional effect. A pharmacological approach was employed, based on the use of 2 µM TG (to deplete ER), 20 µM Bafilomycin A1 (Baf-A1, to affect lysosomes) and 1 µM FCCP (to uncouple mitochondria). All the experiments were performed in 0 Ca_out_ by the use of two different protocols, as shown in Figure 4. Quantification included three parameters as major descriptors of calcium release: peak amplitude, area and the decay tau of the calcium spikes. Cells pretreated with TG drastically decreased the responses to ATP as well as to UTP, suggesting a major contribution for ER (Figure 4A,D–I for quantification): however, a small TG-insensitive component could be detected, suggesting that other stores may be recruited by ATP and UTP. Pretreatment with FCCP or Baf-A1 affected the features of both the spikes induced by ATP or UTP (Figure 4B–I for quantification). Preincubation with the ionophore Ionomycin (Iono, 2 µM) completely prevented the response to ATP. Moreover, the application of a second experimental protocol revealed the complete abolition of the [Ca^2+^]c increase induced by TG and Baf-A1 in cells pretreated with ATP or UTP, globally indicating an interplay among ER, mitochondria and lysosomes in the Ca^2+^ release triggered by both the purinergic agonists (Figure 4J–L).

## 3. Discussion

We have recently shown that high non-physiological eATP doses (>20 µM) strongly inhibit migration of ECs from human breast carcinoma (BTEC) and enhances in vitro endothelial normalization [36]. These events could be related to an overall potential anti-angiogenic activity of strong purinergic stimulation in cancer. Although purinergic signals have been deeply investigated in tumor microenvironment [40], few data are available on ATP-mediated Ca^2+^ signals in TECs.

For these reasons, we investigated ATP-mediated Ca^2+^ signals and their role on migration in both breast and renal-derived ECs.

Here we show that high ATP stimulation (100 μM) triggers biphasic Ca^2+^ signals in BTEC and RTEC; these data are in accordance with the expression of both metabotropic and ionotropic receptors previously reported [36]. In particular, ATP mediates an initial transient [Ca^2+^]c rise due to Ca^2+^ mobilization from intracellular stores, followed by a long-lasting plateau due to Ca^2+^ entry through the plasma membrane. Considering the recruitment of both metabotropic and ionotropic ATP receptors, the long-lasting phase could be due to both store-operated calcium entry (SOCE) and/or non-SOCE mechanisms. SOCE is clearly observed both in BTEC and RTEC when stimulated with 30 µM CPA or TG, two widely used ER depletors. On the other hand, the pharmacological inhibition of SOCE with 20 µM BTP-2 significantly prevented ATP-mediated Ca^2+^ entry in BTEC but did not completely abolish the plateau phase, revealing a non-SOCE component of Ca^2+^ entry induced by 100 μM ATP, presumably related to P2RX-ionotropic receptors.

A section of the paper was devoted to correlate ATP-mediated Ca^2+^ signals with BTEC migration. The ability of 100 µM ATP to decrease BTEC migration even in the presence of BTP-2 unveils a SOCE-independent component in the anti-migratory effect of ATP, possibly related to the recruitment of P2XR-ionotropic receptors that act as calcium-permeable channels [30].

Intracellular calcium stores globally affect BTEC migration since the treatment with CPA or TG, two ER-calcium depletors, as well as 2-APB, remarkably impairs BTEC migration; this evidence points to a general involvement of ER-related calcium release in the regulation of constitutive BTEC migration; nonetheless, and intriguingly, the ability of ATP to retain its antimigratory activity even in TG-preconditioned cells indicates that at least a significant component of ATP-induced functional effects is independent of the release of calcium from ER. The idea that calcium stores could be necessary but not selectively required to sustain the antimigratory action of high eATP is further strengthened by the failure of 100 µM UTP, that actually triggers a calcium release similar to that measured upon ATP stimulation, to promote the same functional effect as ATP. Moreover, a detailed quantitative pharmacological investigation by the use of drugs that selectively impair ER, mitochondria and lysosomes led us to support an interplay among these intracellular organelles in the shaping of purinergic-related calcium release in BTEC: however, no dramatic differences were detected in the quantitative features of this process comparing responses to ATP, that inhibits migration, and UTP, that fails to exert the same functional effect.

Other purinergic agonists such as Adenosine and ADP, respectively acting on P1 and some P2YRs, are able to interfere with TEC migration [36]: here we show that both of them elicit calcium signals.

Taken together, our data suggest that SOCE and calcium release from intracellular stores are not strictly and selectively required for the anti-migratory action of ATP on human tumoral endothelium.

Future investigation will shed light on the involvement of other mechanisms, including store independent calcium entry directly supported by P2XR ionotropic receptors, or Ca^2+^-independent pathways, as recently reported for TRPM8 in endothelial cells [41].

## 4. Materials and Methods

### 4.1. Cell Cultures

Breast tumor-derived endothelial cells (BTEC) and Renal tumor-derived endothelial cells (RTEC) from human breast lobular-infiltrating carcinoma biopsy and renal carcinoma, respectively, were isolated and are periodically characterized in the laboratory of Professor Benedetta Bussolati (Department of Molecular Biotechnology and Health Sciences, University of Torino, Italy) [42].

BTEC and RTEC were grown in EndoGRO-MV-VEGF (Merck Millipore, Burlington, MA, USA) as previously described [36].

### 4.2. Calcium Imaging and Experimental Protocols

Cells were grown on glass gelatin-coated coverslips (gelatin-coating was avoided for experiments using Tyrode physiologic solution without Ca^2+^) at a density of 5000 cells/cm2 for 24–48 h. Cells were next loaded (45 min at 37 °C) with 2 μM Fura-2AM (Invitrogen, Carlsbad, CA, USA), for ratiometric cytosolic Ca^2+^ [Ca^2+^]c measurements. Fluorescence was acquired by Nikon Eclipse TE-2000S (Minato, Tokyo, Japan) inverted microscope. [Ca^2+^]c was expressed as a ratio (R) of emitted fluorescence at 510 nm corresponding to excitation wavelengths of 340 nm and 380 nm.

Metafluor Imaging System (Molecular Devices, Sunnyvale, CA, USA) was used for image acquisition (3 s frequency). For each experiment, several regions of interest (ROIs) have been selected corresponding to single cells in the chosen image field. Real time background subtraction was applied in order to limit noise. Average traces shown in figures include at least 20 ROIs.

Calcium imaging analysis and quantification (peak amplitude, area, rise time, decay time) were performed using Clampfit software (Axon PClamp, Molecular Devices, San Jose, CA, USA) and analysed with GraphPad Prism 7 (GraphPad Software, Inc., La Jolla, CA, USA).

Area underlying calcium influx during the sustained phase in the presence of extracellular Ca^2+^ was evaluated at 300 s after the onset of the response. Total area under calcium spikes in 0 Ca_out_ was measured by the use of Event Detection protocol in Clampfit software.

Ca^2+^ store depletion and store-operated Ca^2+^ entry (SOCE) were evaluated by exploiting the Ca^2+^ add-back protocol. Briefly, the cells were treated with the agonist to induce depletion of Ca^2+^ stores in Ca^2+^-free medium (0 Ca_out_; Tyrode solution without CaCl_2_ added with 2 mM Ethylene Glycol Tetraacetic Acid, EGTA) and, subsequently, replaced with Ca^2+^-containing solution (2 mM Ca^2+^) so that SOCE could be measured.

### 4.3. Migration Assays

Scratch Wound Healing Assay. Cells were grown to confluence on 24-well culture plates. Cell monolayers were allowed to rest for 12 h in EndoGro-MV and a wound was made by scraping the middle of the cell monolayer with a P10 pipette tip. Floating cells were removed by washing twice with Phosphate-Buffered Saline, PBS, and the cell monolayer was put in a serum-free RPMI medium for 1 h and then in RPMI 10% FBS.

Cells did not undergo any significant degree of mitosis during the experiments. Images were acquired using a Nikon Eclipse Ti-E microscope with a 4X objective. Cells were kept at 37 °C and 5% CO_2_ and pictures were taken every 2 h using Metamorph software (Molecular Devices, Sunnyvale, CA, USA) [43,44]. Migration was measured up to 8 h with Metamorph software and expressed as percentage of maximal migration [45,46]. At least three fields for each condition were analyzed in each independent experiment. At least three independent experiments were done for each experimental condition.

### 4.4. RNA Extraction and Real-Time PCR Analysis

Total RNA was extracted from cell samples using PureLink^®^ RNA Mini Kit (ThermoFisher Scientific, Waltham, MA, USA). For quantitative real-time polymerase chain reaction (qRT-PCR), 0.5–1 μg total RNA was transcribed into complementary DNA (cDNA) by High-Capacity cDNA Reverse Transcription Kit (Applied Biosystems, Foster City, CA, USA) upon treatment with RQ1 RNase-Free DNase (Promega, Madison, WI, USA). qRT-PCR was performed using the QuantStudio™ 6 Flex Real-Time PCR System (Applied Biosystems). Primers and probes were designed using the Universal ProbeLibrary Assay Design Center software (www.lifescience.roche.com). Transcript abundance, normalized to 18 s mRNA expression, is expressed as a fold increase over a calibrator sample.

### 4.5. Statistical Analysis

Data were analyzed with GraphPad Prism 7 (GraphPad Software, Inc., La Jolla, CA, USA). Each experiment was repeated at least three times. Preliminary Shapiro-Wilk test was performed to check the normal distributions of each dataset: accordingly, statistical analysis was performed by using either the non-parametric Mann-Whitney test or the Student’s t-test. A *p*-value of <0.05 was considered significant.

## 5. Conclusions

Purinergic stimulation in BTEC evokes different Ca^2+^ signals according to the agonist and its concentration. High eATP doses strongly inhibit BTEC [36] and RTEC migration and trigger biphasic Ca^2+^ signals due to Ca^2+^ release from intracellular stores (mainly ER) and Ca^2+^ entry from extracellular medium. Similar calcium signals and antimigratory effects are triggered by ADP and Adenosine, while P2RY-selective agonist UTP promotes calcium signals without any detectable modulation of TEC migration. Calcium release from ER is not required to account for the entire antimigratory activity of high ATP, while SOCE is not significantly involved. Non-SOCE mechanisms, presumably related to P2RXs recruitment, could be good candidates as negative regulators of TEC migration, but calcium-independent pathways cannot be excluded.

## Supplementary Materials

The following are available online at https://www.mdpi.com/2072-6694/11/6/766/s1, Figure S1: Characterization of high ATP stimulation in RTEC. Figure S2: P1 receptors are expressed and functional in BTEC.
