# Supplementary figures and images for: Purinergic Calcium Signals in Tumor-Derived Endothelium
Source: Cancers (Basel). 2019 Jun 1;11(6):766. doi: 10.3390/cancers11060766 (PMC6627696; doi:10.3390/cancers11060766)

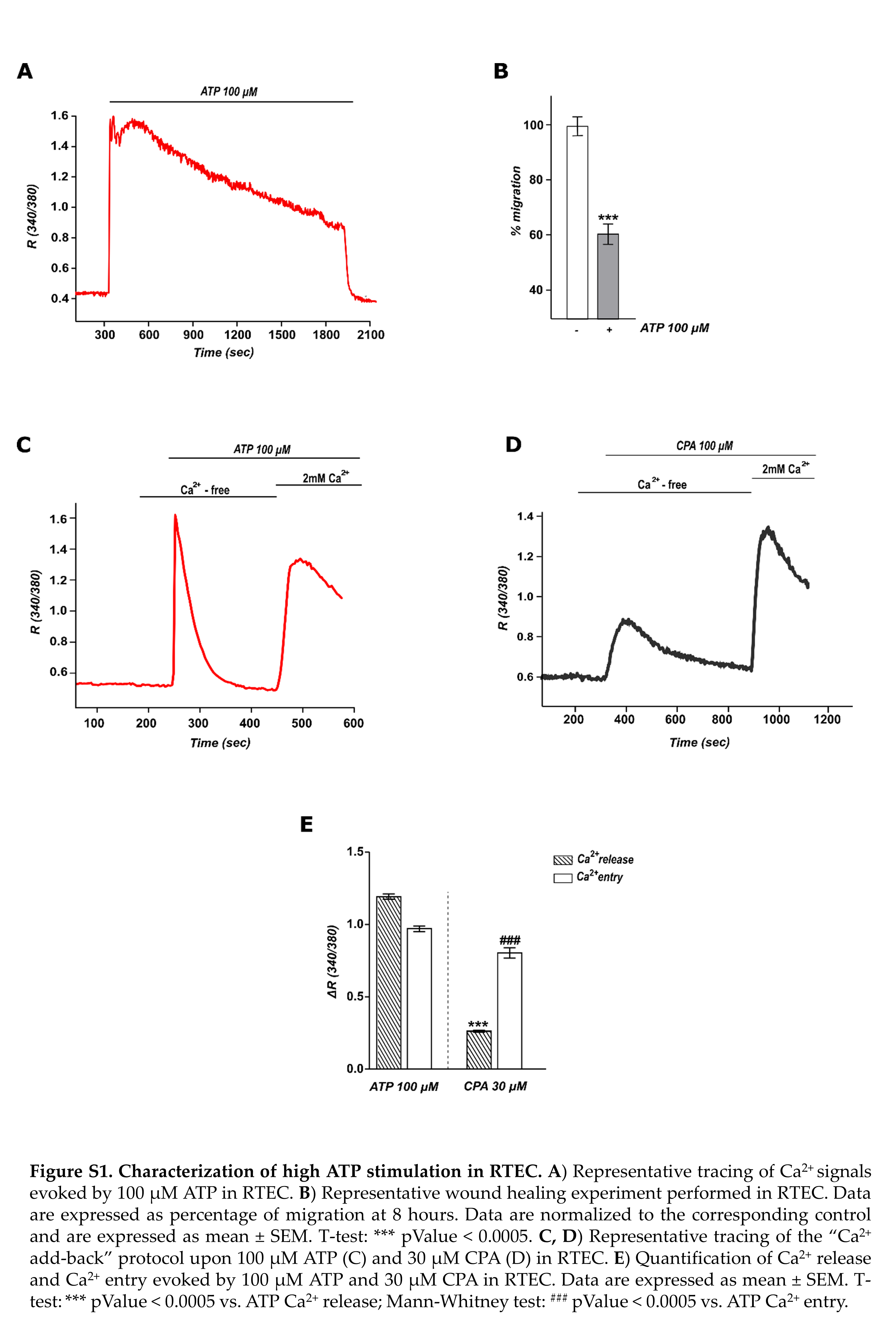

Supplement: Supplementary file 1 [file cancers-11-00766-s001.zip › Supplementary/Figure S1.tif]

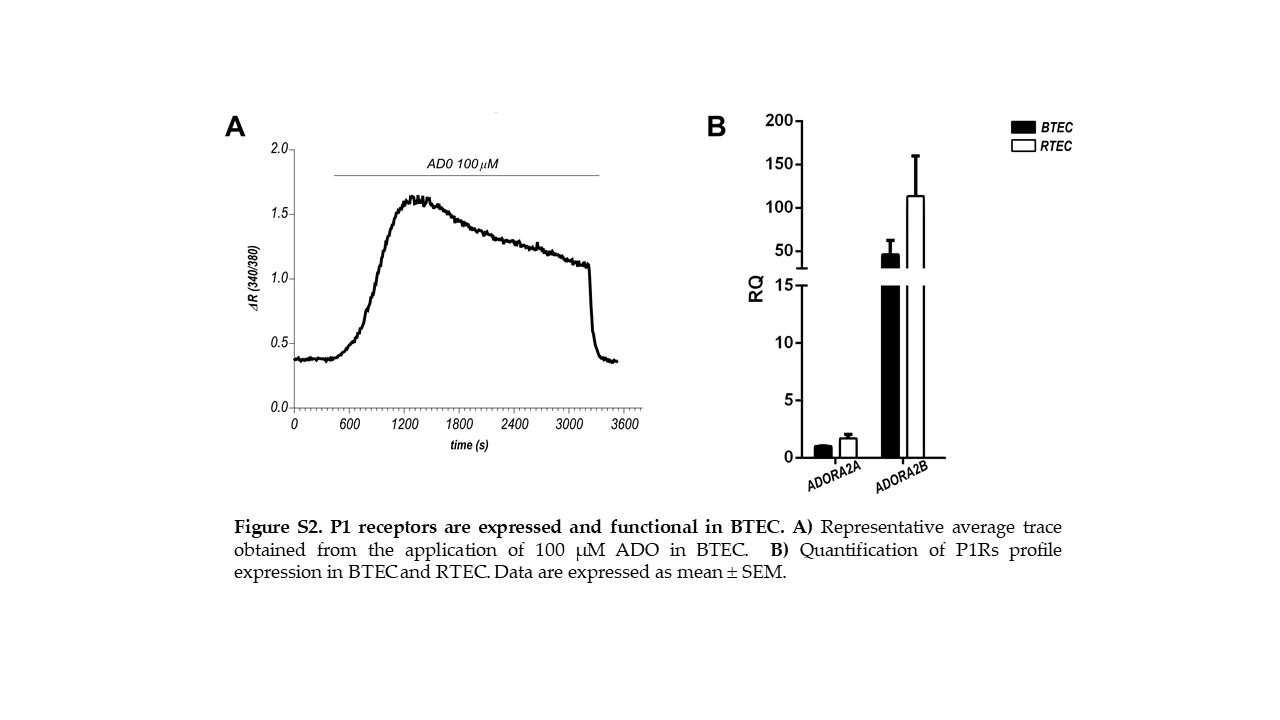

Supplement: Supplementary file 1 [file cancers-11-00766-s001.zip › Supplementary/Figure S2.tif]
